# Effect of Anticoagulation and Individualized Fluid Therapy on Graft Outcomes in Simultaneous Pancreas-Kidney Transplant Recipients

**DOI:** 10.7759/cureus.96728

**Published:** 2025-11-12

**Authors:** Maria E Batista, Miguel Barbosa, Pedro Oliveira, Tiago Isidoro Duarte, José Casimiro, Sofia Cardoso, Nuno Germano

**Affiliations:** 1 Department of Critical Care Medicine, Hospital Curry Cabral, Unidade Local de Saúde de São José, Lisbon, PRT; 2 Department of Intensive Care, Hospital Curry Cabral, Unidade Local de Saúde de São José, Lisbon, PRT; 3 Department of Critical Care Medicine, Hospital de São José, Unidade Local de Saúde de São José, Lisbon, PRT

**Keywords:** anticoagulation, fluid management, graft survival, simultaneous pancreas-kidney transplantation (spk), thrombosis, transpulmonary thermodilution, unfractioned heparin

## Abstract

Background

Simultaneous pancreas-kidney (SPK) transplantation is the preferred treatment for type 1 diabetes mellitus with end-stage renal disease. Early graft loss, especially from pancreatic thrombosis, remains a major challenge. Optimal anticoagulation and fluid strategies are critical but not standardized.

Methodology

We retrospectively reviewed 64 SPK transplants in our center. Group 1 (n = 28) received liberal fluid infusion guided by urine output (>200 mL/hour) and prophylactic low-molecular-weight heparin. Group 2 (n = 36) received unfractionated heparin (UFH) infusion titrated to an activated partial thromboplastin time ratio of 1.5 and PiCCO®-guided fluid management, as a new postoperative protocol was implemented. Patient and graft characteristics and outcomes were analyzed.

Results

Donor and recipient baseline characteristics were comparable. We found a meaningful reduction in pancreas graft thrombosis in Group 2 (6% vs. 18%, p = 0.167), along with similar hemorrhagic complications (21% vs 28%, p = 0.187). Group 2 had higher cumulative fluid balance at 72 hours (9,704 vs. 7,436 mL, p = 0.027) and improved P/F ratio on day 3 (304 vs. 296, p = 0.033). We also documented a reduction in renal delayed graft function (11% vs. 3%, p = 0.171). A relevant increase in pancreas graft survival was seen (71% vs. 92%, p = 0.641), but renal graft survival was similar. Hospital and intensive care unit mortality was similar between the two groups.

Conclusions

UFH anticoagulation combined with PiCCO®-guided fluid management may reduce pancreas thrombosis and delayed renal graft function, without significantly increasing bleeding risk. A patient-specific perioperative approach could enhance SPK transplant outcomes.

## Introduction

Simultaneous pancreas-kidney (SPK) transplantation has emerged as the gold standard treatment for type 1 diabetes mellitus (T1DM) patients with end-stage renal disease. A successful SPK transplantation restores optimal glycemic control, thus halting the progression of other T1DM secondary complications such as diabetic retinopathy and neuropathy, cardiovascular disease, and life-threatening hypoglycemic episodes [1-3]. The majority of large-scale studies agree that SPK is superior to living donor kidney or deceased donor kidney transplantation in terms of donor survival and quality of life [4,5]. This is primarily attributed to advances in surgical techniques and immunosuppressive regimens, resulting in pancreas graft survival rates of 87% at one year and 75% at five years [6]. Secondarily, control of optimal fluid balance is an essential part of organ perfusion management, namely, in solid organ transplants. Traditionally, a high positive fluid balance was implemented to ameliorate renal graft function; however, there is limited evidence supporting this practice [7-9].

Despite significant progress, SPK transplantation remains a challenging procedure, with substantial risks of graft failure as well as recipient morbidity and mortality [10]. Pancreatic graft thrombosis continues to be one of the most common early surgical complications, reported in 4-20% of cases, and is the primary reason for reoperation and early graft loss [11,12]. Multiple risk factors have been described, but the underlying mechanisms are not yet completely understood [12]. At present, there is no universally accepted protocol for thrombosis prevention, although most centers use some form of anticoagulation. In 2021, we shared preliminary results from our cohort [13]; here, we provide an updated retrospective analysis with a larger patient population, assessing the safety and efficacy of our postoperative strategy, which combines anticoagulation with individualized fluid management guided by a standardized algorithm.

This article was previously presented as a poster at Lives 2023, the 36th Annual Congress of the European Society of Intensive Care Medicine, on October 22, 2023.

## Materials and methods

Patients who underwent SPK transplant at Hospital Curry Cabral were included in the study. The Local Ethical Committee approved this study and its publication (approval number: 1621/2025). Written informed consent was waived by the Ethics Committee. Data were collected retrospectively over six years, and patients were divided into two groups based on the implementation of the new postoperative protocol in our intensive care unit. Group 1 (n = 28) included SPK transplants who received liberal continuous fluid infusion based on urine output >200 mL/hour and prophylactic low-molecular-weight heparin (LMWH). Group 2 (n = 36) included SPK transplants under the new protocol, whose fluid balance was guided by transpulmonary thermodilution monitoring (PICCO®) and received unfractionated heparin (UFN) infusion.

All patients started acetylsalicylic acid 100 mg/day preoperatively and continued it after the transplant. After the transplant, all patients were admitted to our intensive care unit (ICU) for surveillance and monitoring. Traditionally, all patients also received LMWH after surgery and liberal continuous fluid infusion based on urine output >200 mL/hour. In line with the latest perspectives, we adopted a new protocol in which UFH was initiated immediately after surgery at 500 units per hour and maintained for five days. The infusion rate was reassessed every four hours and adjusted to achieve a target activated partial thromboplastin time (aPTT) ratio of 1.5 (1 unit/kg/hour). With respect to fluid management, balanced electrolyte solutions were administered. Hemodynamic monitoring using pulmonary thermodilution with the PICCO® catheter was performed hourly during the first six postoperative hours, then every four hours until the third postoperative day, after which it was spaced to every eight hours. The PICCO® catheter was typically removed on the sixth day following transplantation.

We utilized global end-diastolic index (GEDI), extravascular lung water index (ELWI), and diuresis to attain the optimal fluid balance (GEDI = 680-800mL/m2; ELWI <10 mL/kg, diuresis >200 mL/hour).

Hemorrhagic complications were defined as having at least three blood transfusions in one day, the need for emergent surgical intervention, and hemorrhagic shock, which led to temporary suspension of anticoagulation therapy.

The objective of this study was to evaluate the impact of anticoagulation regimen and individualized fluid therapy on graft outcomes in SPK transplant recipients. We hypothesized that optimized immediate postoperative anticoagulation and fluid management would improve graft function and reduce postoperative complications.

Surgical procedure

For the surgical approach, a midline incision was performed, followed by complete clamping of the right iliac vein and artery. Venous and arterial anastomoses were created in a vein-to-vein and artery-to-artery fashion. The donor duodenum was anastomosed to a recipient intestinal loop, and the pancreas graft was positioned horizontally.

Immunosuppression

Induction therapy consisted of intravenous methylprednisolone (500 mg) combined with thymoglobulin (1.5 mg/kg) administered before skin incision. From postoperative day two, patients were transitioned to oral prednisolone at 20 mg daily. Mycophenolic acid was introduced on day one at 250 mg intravenously twice daily and converted to 180 mg orally twice daily on day five. Tacrolimus infusion (0.01 mg/kg/day) was initiated within the first 12 hours following ICU admission, adjusted to maintain trough levels around 10 ng/mL, and subsequently changed to oral administration on day five.

Graft follow-up

Laboratory monitoring was performed twice daily during the first 48 hours after transplantation and subsequently once per day to evaluate graft performance. The panel included complete blood count, coagulation parameters (aPTT, prothrombin time), serum glucose, C-peptide, renal and hepatic function tests, and C-reactive protein. Doppler ultrasonography was routinely obtained on postoperative day one and repeated when clinically indicated. In cases where abnormal perfusion was suspected on clinical or ultrasound grounds, contrast-enhanced CT was undertaken.

Statistical analysis

Continuous variables are presented as medians with interquartile ranges, while categorical variables are expressed as frequencies and percentages. Group comparisons were performed using the Mann-Whitney U test for continuous data and the chi-square test for categorical data. Pancreas graft survival was estimated using the Kaplan-Meier method. A p-value below 0.05 was considered statistically significant. All analyses were conducted using SPSS software, version 20.0 (IBM Corp., Armonk, NY, USA).

## Results

A total of 64 patients were enrolled in this study: 28 in Group 1 and 36 in Group 2. Regarding recipients’ characteristics, there were no differences in age, gender, body mass index, duration of T1DM, or dialysis. None of the patients had been previously submitted to a transplant. Donor characteristics, represented in Table 1, were similar between the groups. All patients received grafts from neurologic death donors.

**Table 1:** Donor and recipient characteristics. CIT: cold ischemia time; T1DM: type 1 diabetes mellitus; RRT: renal replacement therapy; WIT: warm ischemia time

	Group 1	Group 2	P-value
Recipient characteristics			
Age (years)	37 (10)	39 (14)	0.208
Gender (male/female)	17/11	18/18	0.393
Body mass index (kg/m²)	22.8 (7.1)	24.1 (4.1)	0.461
Duration of T1DM (years)	22 (8)	24 (10)	0.455
Duration of dialysis (months)	22 (16)	23 (18)	0.071
Type of dialysis			
Hemodialysis (%)	19 (68%)	23 (64%)	0.902
Peritoneal dialysis (%)	6 (21%)	10 (28%)	
None (%)	3 (11%)	3 (8%)	
Previous thrombotic events (%)	5 (17.9%)	5 (13.9%)	0.700
Donor characteristics			
Age (years)	42 (18)	31 (25)	0.053
Body mass index (kg/m²)	25.3 (4.1)	23.0 (3.7)	0.283
Previous thrombotic events (%)	3 (10.7%)	3 (8.3%)	0.757
Kidney CIT (minutes)	519 (135)	544 (160)	0.097
Kidney WIT (minutes)	35 (11)	29 (8)	0.173
Pancreas CIT (minutes)	420 (75)	468 (203)	0.123
Pancreas WIT (minutes)	35 (9)	33 (10)	0.662

**Figure 1:**
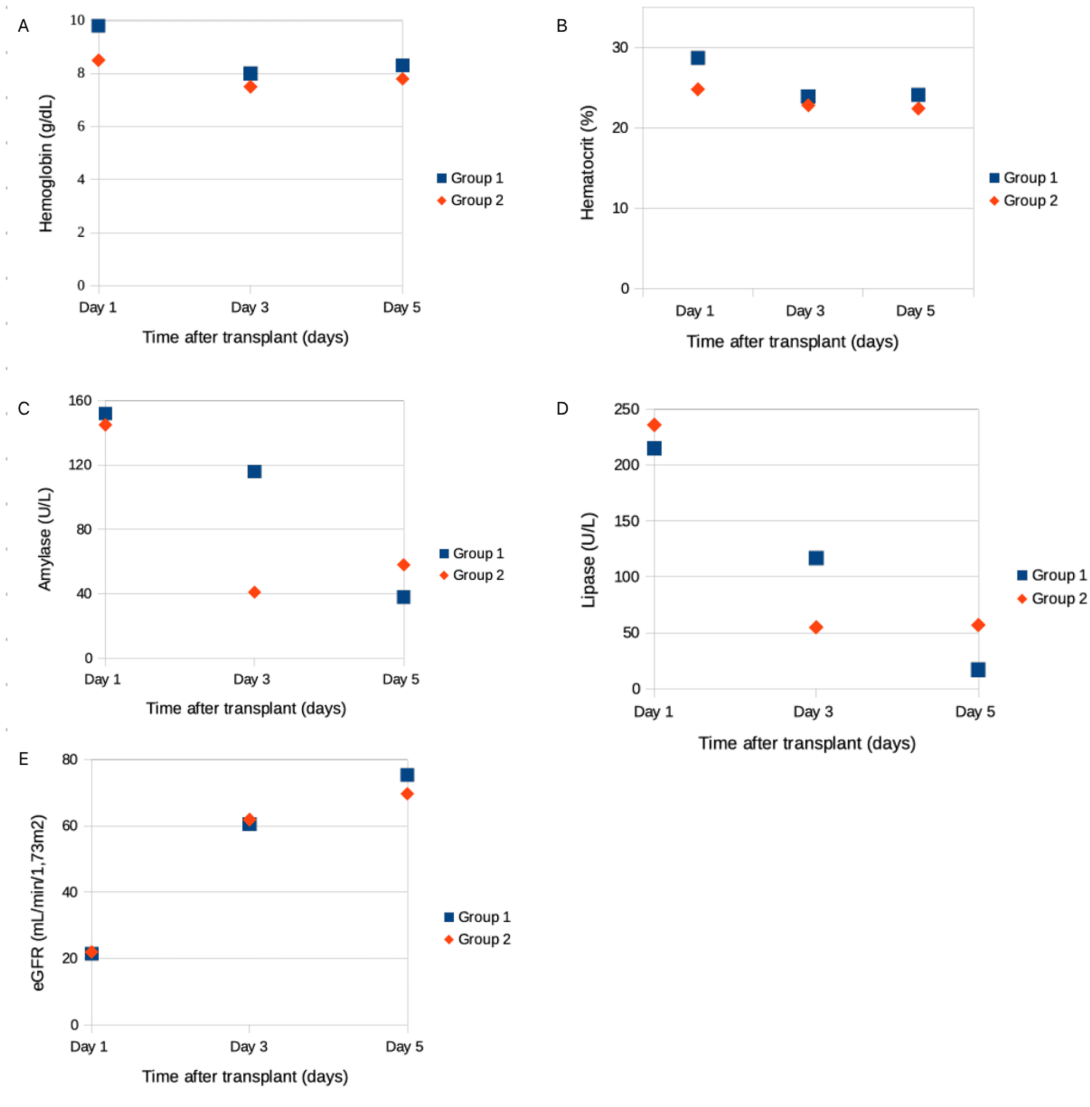
Variation of hemoglobin (A), hematocrit (B), serum amylase (C), serum lipase (D), and eGFR (E) over five days in patients submitted to simultaneous pancreas-kidney transplantation. Values are expressed as median. eGFR: estimated glomerular filtration rate

Table 2 highlights the complication rates found in the postoperative period. Hemorrhagic complications were similar between the two groups (21% vs. 28%; p = 0.187). We noted one gastrointestinal hemorrhage, one hemorrhage from the surgical incision, and 14 hemoperitoneum cases, of which 56% (n = 9) required exploratory laparotomy. The median day of hemorrhage was comparable (day two vs. four; p = 0.111), as well as the number of packed red blood cells. An important decrease in pancreas graft thrombosis was found in Group 2 (18% vs. 6%, p = 0.167), as well as a reduction in renal graft thrombosis (7% vs. 6%, p = 0.817). Regarding biochemical values, hemoglobin and hematocrit levels were higher in Group 1 on the first postoperative day, after which they followed a similar trend in both groups (Table 3, Figure 1).

**Table 2:** Postoperative complications regarding the anticoagulation protocol. *: This value is constant.

	Group 1	Group 2	P-value
Hemorrhage (%)	6 (21%)	10 (28%)	0.309
Day of hemorrhage	1.8 (2)	4.3 (9)	0.111
Number of packed red blood cells	3.5 (3)	2.0 (2.5)	0.344
Number of re-laparotomies (%)	4 (14%)	5 (14%)	0.869
Pancreas graft thrombosis (%)	5 (18%)	2 (6%)	0.167
Day of pancreas graft thrombosis	3	5*	-
Kidney graft thrombosis (%)	2 (7%)	2 (6%)	0.817
Day of kidney graft thrombosis	19	24	0.893

**Table 3:** Biochemical parameters evolution after the transplant. eGFR: estimated glomerular filtration rate

	Group 1	Group 2	P-value
Day 1 Hemoglobin (g/dL)	9.8	8.5	0.009
Day 3 Hemoglobin (g/dL)	8.0	7.5	0.219
Day 5 Hemoglobin (g/dL)	8.3	7.8	0.130
Day 1 Hematocrit (%)	28.7	24.8	0.020
Day 3 Hematocrit (%)	23.9	22.8	0.400
Day 5 Hematocrit (%)	24.1	22.4	0.082
Day 1 Serum amylase (U/L)	152	145	0.909
Day 3 Serum amylase (U/L)	116	41	0.033
Day 5 Serum amylase (U/L)	38	58	0.235
Day 1 Serum lipase (U/L)	215	236	0.946
Day 3 Serum lipase (U/L)	117	55	0.442
Day 5 Serum lipase (U/L)	17	57	0.032
Day 1 eGFR (mL/minute/1.73m²)	21.5	22.0	0.885
Day 3 eGFR (mL/minute/1.73m²)	60.5	61.9	0.912
Day 5 eGFR (mL/minute/1.73m²)	75.3	69.7	0.666

Regarding fluid balance management with the PICCO catheter, we noted a higher mean fluid balance in the first 24 and 48 hours in Group 1 (4,172 mL vs 3,441 mL, p = 0.169; 2,945 mL vs. 2,839 mL, p = 0.853). On the third day, there was a different trend; fluid balance was greater in Group 2 (691 mL vs 1,295 mL, p = 0.236). The mean cumulative fluid balance in the first 72 hours was significantly higher in Group 2 (7,436 mL vs. 9,704 mL, p = 0.027). The mean P/F ratio was similar between the two groups at 24 and 48 hours (447 vs. 376, p = 0.766; 336 vs. 343, p = 0.810), whereas on the third day, it was significantly higher in Group 2 (296 vs. 304, p = 0.033). No significant differences were found in glomerular filtration rate (Table 4, Figure 1). Renal delayed graft function had a meaningful decrease in Group 2 (11% vs. 3%, p = 0.171); multivariate analysis revealed that renal cold ischemia time did not predict delayed graft function.

**Table 4:** Postoperative comparison of the fluid balance management protocol.

	Group 1	Group 2	P-value
Day 0 fluid balance (mL)	4,320	4,087	0.827
Day 1 fluid balance (mL)	4,172	3,441	0.169
Day 2 fluid balance (mL)	2,944	2,838	0.853
Day 3 fluid balance (mL)	691	1,295	0.236
Cumulative fluid balance (mL)	7,436	9,704	0.027
Day 1 P/F ratio	447	376	0.766
Day 2 P/F ratio	336	343	0.810
Day 3 P/F ratio	296	304	0.033

We documented a relevant increase in pancreas graft survival (71% vs. 92%, p = 0.641) and similar renal graft survival. Hospital and ICU mortality were similar between the two groups (Table 5).

**Table 5:** Graft and patient survival. ICU: intensive care unit

	Group 1	Group 2	P-value
Infection during ICU (%)	1 (4%)	14 (34%)	0.114
ICU death (%)	1 (4%)	1 (3%)	0.683
Hospital death (%)	0	2 (6%)	0.276
Renal delayed graft function (%)	3 (11%)	1 (3%)	0.171
Pancreas graft survival (%)	20 (71%)	33 (92%)	0.641
Kidney graft survival (%)	24 (86%)	31 (86%)	0.369
One-year pancreas graft survival (%)	19 (68%)	32 (89%)	0.096
One-year kidney graft survival (%)	22 (79%)	30 (83%)	0.770
One-year basal creatinine (mg/dL)	1.28	1.41	0.492

**Figure 2:**
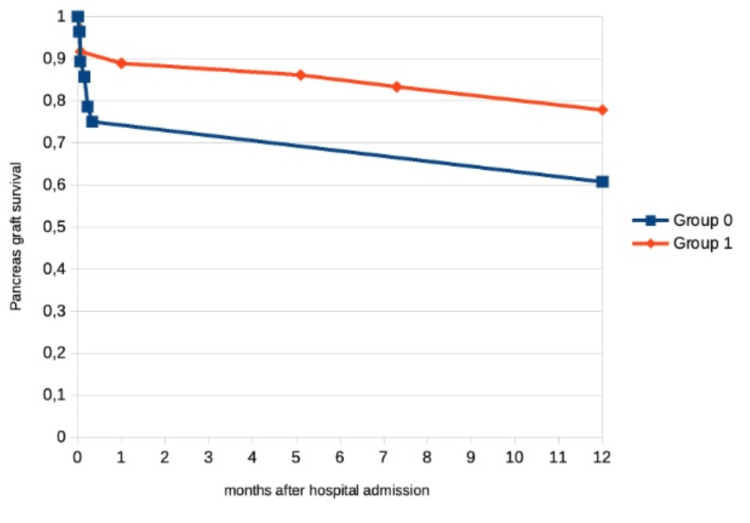
Pancreas graft survival following simultaneous pancreas-kidney transplantation.

**Figure 3:**
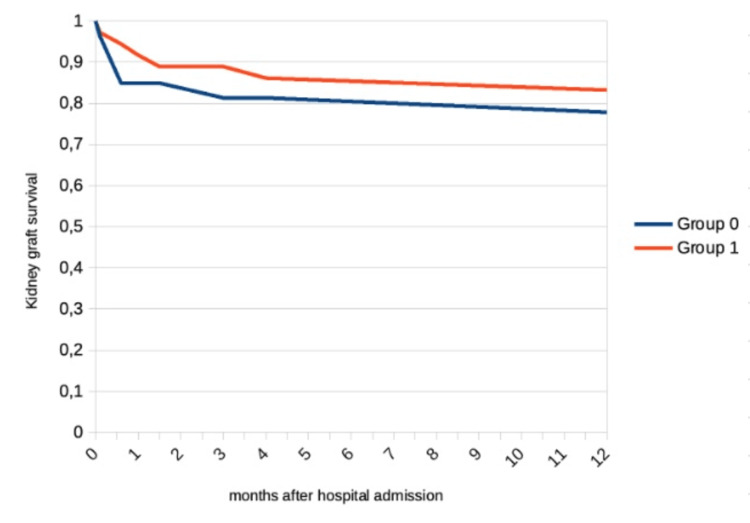
Kidney graft survival following simultaneous pancreas-kidney transplantation.

Regarding one-year graft survival, we noted a marked increase in pancreas graft survival (68% vs. 89%, p = 0.096) and similar kidney graft survival (79% vs. 83%, p = 0.770), as seen in Figure 2 and Figure 3. One year after transplant, basal creatinine was similar between the groups (1.28 vs. 1.41, p = 0.492).

## Discussion

Given the intrinsically low parenchymal microvasculature flow of the pancreas and the hypercoagulable state of the diabetic recipient, these patients are at a higher risk of graft thrombosis than other abdominal organ transplants [11]. These factors, combined with the inevitable hemodynamic changes that occur during transplantation, such as reperfusion injury, cellular necrosis, tissue edema, and increased local vascular resistance, constitute significant risk factors for graft thrombosis and ischemia-related pancreatitis [10,14].

Published approaches to prevention are heterogeneous. Several observational series report a protective association between early low-dose heparin and reduced rates of pancreatic graft thrombosis. Humar et al. used a low dose of UFH for five days and acetylsalicylic acid for three months and reported a significant reduction in pancreas graft thrombosis (12.1% vs. 5.8%; p = 0.02), although there was a small increase in bleeding complications requiring laparotomy (4.9% vs. 6.6%; p = 0.14) [15]. Similarly, Scheffert et al. used low-dose UFH infusion in the early postoperative period (500 UI/hour for two days) and reported a reduction in thrombotic events despite not achieving statistical significance [16]. Aboalsamh et al. compared smaller doses of UFH with acetylsalicylic acid alone and described a reduction in graft thrombosis as well as improved graft survival, with no differences in complication rates [17]. Instead, Schenker et al. compared low-dose UFH to once-daily fixed dose of LMWH and concluded that LMWH was non-inferior to UFH in preventing pancreas graft thrombosis with similar complications as major bleeding requiring laparotomy [18]. Raveh et al. further suggested that a susceptibility-directed anticoagulation strategy can reduce thrombotic complications by tailoring prophylaxis to individual patient risk [19].

Conversely, Okabe et al. reported favorable outcomes without routine pharmacologic thromboprophylaxis, attributing low thrombosis rates to meticulous intra- and postoperative fluid management and surgical technique rather than to systematic anticoagulation [20]. Instead, they aimed for a hematocrit of 25%, and albumin products were given to maintain intravascular fluid volume. In addition, they relied on the frequent monitoring of graft blood flow through ultrasound evaluation. Their findings illustrated that optimal volume status and graft perfusion can, in some settings, mitigate thrombosis risk even in the absence of routine heparinization.

Despite the absence of a standardized anticoagulation regimen across transplant programs, most institutions now employ some form of postoperative heparinization to mitigate thrombosis risk. In line with these findings, our results indicate that continuous UFH infusion was associated with improved graft survival, albeit with a higher rate of minor hemorrhagic complications. These observations support the growing consensus that the benefits of early anticoagulation may outweigh the risks when carefully titrated and monitored.

Additionally, fluid management is an important factor known to affect delayed graft function and long-term transplant outcomes. The difficulty lies in achieving the best preload and graft perfusion without the deleterious effect of hypervolemia. Our six-year cohort reports an important decrease in delayed renal graft function, with higher P/F ratios, suggesting that PiCCO® monitoring might be beneficial to attain the optimal fluid balance tailored for each patient.

The main limitation of our study is its retrospective design. Additionally, the small cohort of patients has limited statistical power. The surgical team remained the same, and none changed their surgical technique over time. Although it was not described in this study, intraoperative management must be included in future studies as it may influence graft survival.

## Conclusions

In this cohort of SPK transplant recipients, individualized postoperative management, encompassing tailored anticoagulation and goal-directed fluid therapy, was associated with improved graft function and a similar incidence of thrombotic events. These results highlight the value of a comprehensive, patient-centered perioperative strategy that integrates risk assessment, continuous monitoring, and individualized therapeutic adjustments to optimize graft survival.
